# Circulating CCL20 as a New Biomarker of Abdominal Aortic Aneurysm

**DOI:** 10.1038/s41598-017-17594-6

**Published:** 2017-12-11

**Authors:** B. Soto, T. Gallastegi-Mozos, C. Rodríguez, J. Martínez-González, J.-R. Escudero, L. Vila, M. Camacho

**Affiliations:** 1 Angiology, Vascular Biology and Inflammation Laboratory, Biomedical Research Institute Sant Pau, (IIB-Sant Pau), Barcelona, Spain; 2 Servicios Mancomunados de Angiología, Cirugía Vascular y Endovascular, Hospitales de la Santa Creu i Sant Pau/Dos de Mayo, Universitat Autonoma de Barcelona, Biomedical Research Institute Sant Pau (IIB Sant Pau), Barcelona, Spain; 3 Institut Català de Ciéncies Cardiovasculars (ICCC), Biomedical Research Institute Sant Pau (IIB Sant Pau), Barcelona, Spain; 4 CIBER de Enfermedades Cardiovasculares (CIBERCV), Madrid, Spain; 5 Institute of Biomedical Research of Barcelona (IIBB-CSIC), Biomedical Research Institute Sant Pau, Barcelona, Spain

## Abstract

Autoimmunity appears to play a role in abdominal aortic aneurysm (AAA) pathology. Although the chemokine CCL20 has been involved in autoimmune diseases, its relationship with the pathogenesis of AAA is unclear. We investigated CCL20 expression in AAA and evaluated it as a potential biomarker for AAA. CCL20 was measured in plasma of AAA patients (n = 96), atherosclerotic disease (AD) patients (n = 28) and controls (n = 45). AAA presence was associated with higher plasma levels of CCL20 after adjustments for confounders in the linear regression analysis. Diagnostic performance of plasma CCL20 was assessed by ROC curve analysis, AUC 0.768 (CI:0.678–0.858; p<0.001). Classification and regression tree analysis classified patients into two CCL20 plasma level groups. The high-CCL20 group had a higher number of AAA than the low-CCL20 group (91% vs 54.3%, p< 0.001). mRNA of CCL20 and its receptor CCR6 were higher in AAA (n = 89) than in control aortas (n = 17, p<0.001). A positive correlation was found between both mRNA in controls (R = 0674; p = 0.003), but not in AAA. Immunohistochemistry showed that CCR6 and CCL20 colocalized in the media and endothelial cells. Infiltrating leukocytes immunostained for both proteins but only colocalized in some of them. Our data shows that CCL20 is increased in AAA and circulating CCL20 is a high sensitive biomarker of AAA

## Introduction

Abdominal aortic aneurysm (AAA) is a progressive focal dilatation and weakening of the abdominal aorta, being the most common arterial aneurysm. AAA affects a high percentage of the aged population in industrialized countries. AAA is a complex multifactorial disease with genetic and environmental risk factors^1^. The disease is progressive, with growth and even rupture^2^. Mortality from ruptured AAA is as high as 90%^3^ and currently open surgery or endovascular repair are the unique available treatment for.

AAA is characterized by chronic inflammation, apoptosis of vascular smooth muscle cells and neovascularisation. Extracellular matrix degradation, microcalcification and oxidative stress are biological processes that contribute to the degeneration of the aorta. The concept of autoimmunity should be taken into account in the AAA pathogenesis and progression, although the precise cause of autoimmunity is not known and the sequence of pathological events and their direct contribution to AAA still remain unexplained (reviewed in^4,5^).

The identification of specific AAA biomarkers that detect high risk patients and monitor disease progression is a challenge. Several serum biomarkers of inflammatory and proteolytic activity have been investigated in patients with AAA including MMPs, interleukins, CRP and other potential mediators such as TNF-α and TIMP-1 that may contribute to connective tissue degradation and collagen and elastin degeneration. However, measurement of plasma concentrations of these biomarkers has not been demonstrated to be of value predicting clinical risk, probably reflecting a lack of sensitivity^6,7^. Proteomic studies have been useful to find new candidate biomarkers^8^ but ultimately plasma concentration could represent the activity of the entire vasculature rather than that of small areas of focal vascular injury. This circumstance coupled with the fact that atherosclerosis in most cases co-exists with AAA, limits the sensitivity and specificity of currently proposed AAA biomarkers. Further, identification of new biomarkers could help to find out new pathways involved in the pathophysiology of AAA and thus to discover new targets for pharmacological intervention. Therefore, it is essential to find new, more specific biomarkers for early detection of the disease and risk stratification.

The C-C chemokine CCL20 (chemokine (C-C motif) ligand 20) is a chemokine expressed mainly in the lymphatic tissue, lung and liver^9–11^ produced by cells related with inflammation and autoimmune response such as endothelial cells, neutrophils, natural killer (NK), Th17 cells and B cells among others^11–19^. It is well established that CCL20 contributes to inflammatory cell recruitment^20^. This chemokine selectively signals through its receptor CCR6 (C-C chemokine receptor type 6) that was originally described in both lymphoid and not lymphoid tissues^11^ and is expressed by several leukocyte subsets, including immature dendritic cells (iDC), B cells, T cells (proinflammatory Th17 cells, regulatory Treg cells), NKT cells and neutrophils^11,21,22^. CCR6 is a key mediator in iDC recruitment during adaptive immune responses^23^. The involvement of CCL20/CCR6 axis in the autoimmune response is sustained by its relation with rheumatoid arthritis, psoriasis and other autoimmune pathologies^17,24–26^. However, to date, the exact role of the CCL20/CCR6 axis in the dynamics of the immune system has yet to be elucidated, despite evidence points to a regulatory role in the balance of activation and suppression of immune and autoimmune responses^17,24^.

The possible contribution of CCL20 to AAA development and progression is currently uncertain. It has been reported an increase in CCL20 levels in plasma samples from AAA patients compared with normal subjects, but the limited number of patients included in this study does not allow to draw definitive conclusions^27^. In view of the foregoing, we have assessed CCL20 expression in AAA patients and compared its prognostic ability with other proteins, which are already postulated as AAA biomarkers.

## Results

### Assessment of plasma levels of pro-inflammatory proteins in AAA and atherosclerotic patients

To study plasma levels of CCL20, 96 patients consecutively admitted to the Angiology and Vascular Surgery Service of our hospital from January 2012 to December 2015 with AAA were included. Also, 28 patients admitted during the same period with diagnosis of ischemic pathology of either lower limbs or carotid territory were included (AD).

Table 1 shows the demographic data of patients included in this study. There were significant differences in the percentage of women, diabetics, patients with peripheral artery disease (PAD), patients with brain vascular disease (BVD), and antiplatelet drug users between both groups of patients. In order to assess whether these variables were confounders, we first analysed them in each group separately. None of these variables influenced statistically CCL20 plasma levels in either group of patients. Afterwards, multiple linear regression analysis was performed in all patients, using plasma levels of CCL20 (logarithmic transformation to normalize the distribution) as the dependent variable and the presence of AAA, sex, diabetes, PAD, BVD and antiplatelet treatment as independent variables. In this case only the presence or absence of AAA significantly influenced the levels of CCL20 (LOG [CCL20] = −0.993 + (0.574 ∗ sex) − (0.292 ∗ diabetes) − (0.118 ∗ PAD) − (0.437 ∗ BVD) + (0.0582 ∗ Anti-platelet) + (1.107 ∗ AAA presence); N = 124, R = 0.439; P of Coefficients: Constant = 0.021, Sex = 0.131, Diabetes = 0.270, PAD = 0.609, BVD = 0.171, Anti-platelet = 0.817 and AAA presence < 0.001). None of these parameters were then taken into account as confounding variables. In addition to CCL20, we analysed plasma levels of other 15 proteins in AAA and AD patients. Plasma levels data did not fit a normal distribution in any case. Results in Table 2 show that there were no statistically significant differences between both groups of patients regarding plasma levels of IL1-β, RANTES, VEGF, MPO, IL-8, soluble ICAM and IP-10. Levels of CCL20, TNFα, MMP-9, IL-2, IGFBP-1, soluble Fractalkine, IL-10, soluble TWEAK were significantly higher in patients with AAA, whereas plasma levels of MMP-2 were significantly higher in AD patients.

**Table 1:** Demographics of individuals included in the plasma levels study.

	AAA	AD	p	Blood donors
n	96	28	—	45
Age (years)	73.2 ± 7.2	71.6 ± 10.3	0.499	63.0 ± 2.9
Women	4.2	28.6	<0.001	13.3
Aortic diameter (mm)	64.7 ± 12.2	—	—	—
Dyslipidemia	52.1	46.4	0.752	2.2
HTN	79.2	89.3	0.350	31.1
Diabetes	20.8	46.4	0.014	2.2
Smokers	25.0	25.0	0.804	13.3
PAD	43.8	75.0	0.007	0.0
BVD	9.4	39.3	<0.001	0.0
IHD	28.1	32.1	0.862	0.0
COPD	16.7	7.1	0.335	0.0
Antiplatelet users	62.5	96.4	0.001	0.0
Statin users	72.9	85.7	0.254	15.6
NSAID users	1.04	0.0	0.509	0.0
Corticoid users	2.1	10.7	0.137	0.0
Immuno-supressors users	3.1	3.6	0.633	0.0

Nominal variables are presented as %. Continuous variables are presented as mean ± SD. p, refers comparison AAA vs AD. BVD, brain-vascular disease; COPD, chronic occlusive pulmonary disease; HTN, chronic hypertension; IHD, ischemic heart disease; PAD, peripheral artery disease.

**Table 2:** Plasma levels of proteins associated with immunoinflammatory response.

Protein	Protein levels (pg/mL)						Ratio	P value
	AAA n = 96			AD n = 28				
	Median	25%	75%	Median	25%	75%		
CCL20	3.16	0.22	6.84	0.11	0.01	0.94	28.73	<0.001
TNFα	7.06	1.475	13.677	1.41	0.69	2.635	5.01	<0.001
MMP-9	49955	29082	71751	15705	12138.5	26944	3.18	<0.001
IL-2	0.25	0.11	2.16	0.13	0.09	0.21	1.92	0.004
IGFBP-1	3.21	2.055	8.455	2.14	1.33	4.72	1.50	0.016
Fractalkine	37.15	26.108	63.995	25.915	14.535	35.555	1.43	<0.001
IL-10	2.03	0.97	8.742	1.52	0.74	2.32	1.34	0.002
TWEAK	0.58	0.422	0.957	0.465	0.35	0.585	1.25	0.007
MMP-2	62221	45878.25	77264.5	84112	66031.5	106996	0.74	<0.001
VEGF	88.47	5.912	244.965	31.23	1.835	219.51	2.83	n.s.
IL1-β	0.63	0.255	2.002	0.32	0.225	0.51	1.97	n.s.
RANTES	9776	4498.75	17181.25	7912.5	5733.5	13704	1.24	n.s.
MPO	14211.9	10603.3	22032.8	14109.9	10312.626	19054.9	1.01	n.s.
IP-10	483.09	372.102	644.22	478.725	378.285	1019	1.01	n.s.
IL-8	3.35	2.115	6.345	3.395	2.28	5.35	0.99	n.s.
ICAM	81204	58957	144202.25	88842.5	74749	106742	0.91	n.s.

Plasma levels of proteins associated with inmunoimmflamatory response were determined in aneurysmal patients (AAA) and patients with atherosclerotic disease (AD). Data did not fit a normal distribution and are expressed as the median with 25 and 75 percentiles. Ratio: median AAA/median AD.

We then performed a multiple logistic regression analysis considering occurrence of AAA as depending dichotomous variable. We included in the study those proteins that were increased in AAA more than two-fold in terms of the median, namely CCL20, TNFα and MMP-9 (see Table 2). There was a statistically correlation between plasma levels of CCL20 and TNFα (R = 0.369, p = 2.5·10^−4^, n = 95) and MMP-9 (R = 0.351, p = 4.9·10^−4^). We then included plasma levels of CCL20, TNFα and MMP-9 as independent variables. Results of the analysis produces the function Logit P = −0.791 + 1.736 ∗ log [CCL20] + 0.0239 ∗ log [TNF] + 0.368 ∗ log [MMP9] (brackets indicate plasma concentration in pg/mL). Details of the Logistic Regression Equation were: [CCL20] coefficient: Wald statistics 6.550 (p = 0.01) and an odds ratio of 5.7 (CI 1.5–21.5); [TNFα] coefficient: Wald statistics 0.00087 (p = 0.976) and an odds ratio of 1.024 (CI 0.21–5.03); for [MMP-9] coefficient: Wald statistics 0.337 and an odds ratio of 1,45 (CI 0.42–5.01). Consequently CCL20 was the only protein later considered in the study.

### Enhanced CCL20 expression in plasma of AAA patients

**Figure 1:**
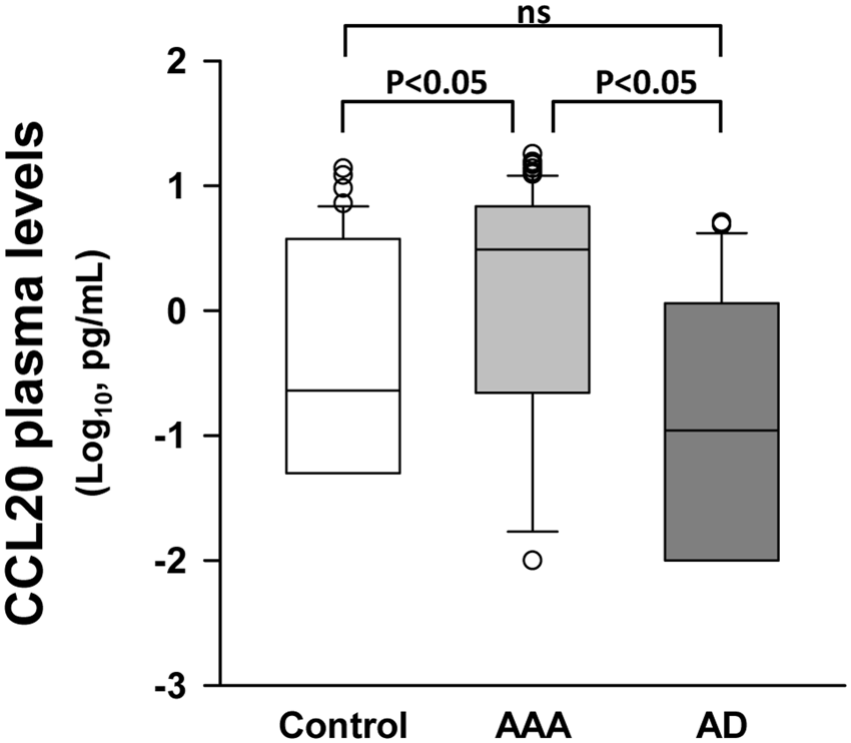
CCL20 plasma concentration in healthy subjects (n = 45), abdominal aortic aneurysm patients (AAA, n = 96) and patients with atherosclerotic disease and without AAA (AD, n = 28). No group fit normal distribution; ns, means no significant differences (p > 0.05).

**Figure 2:**
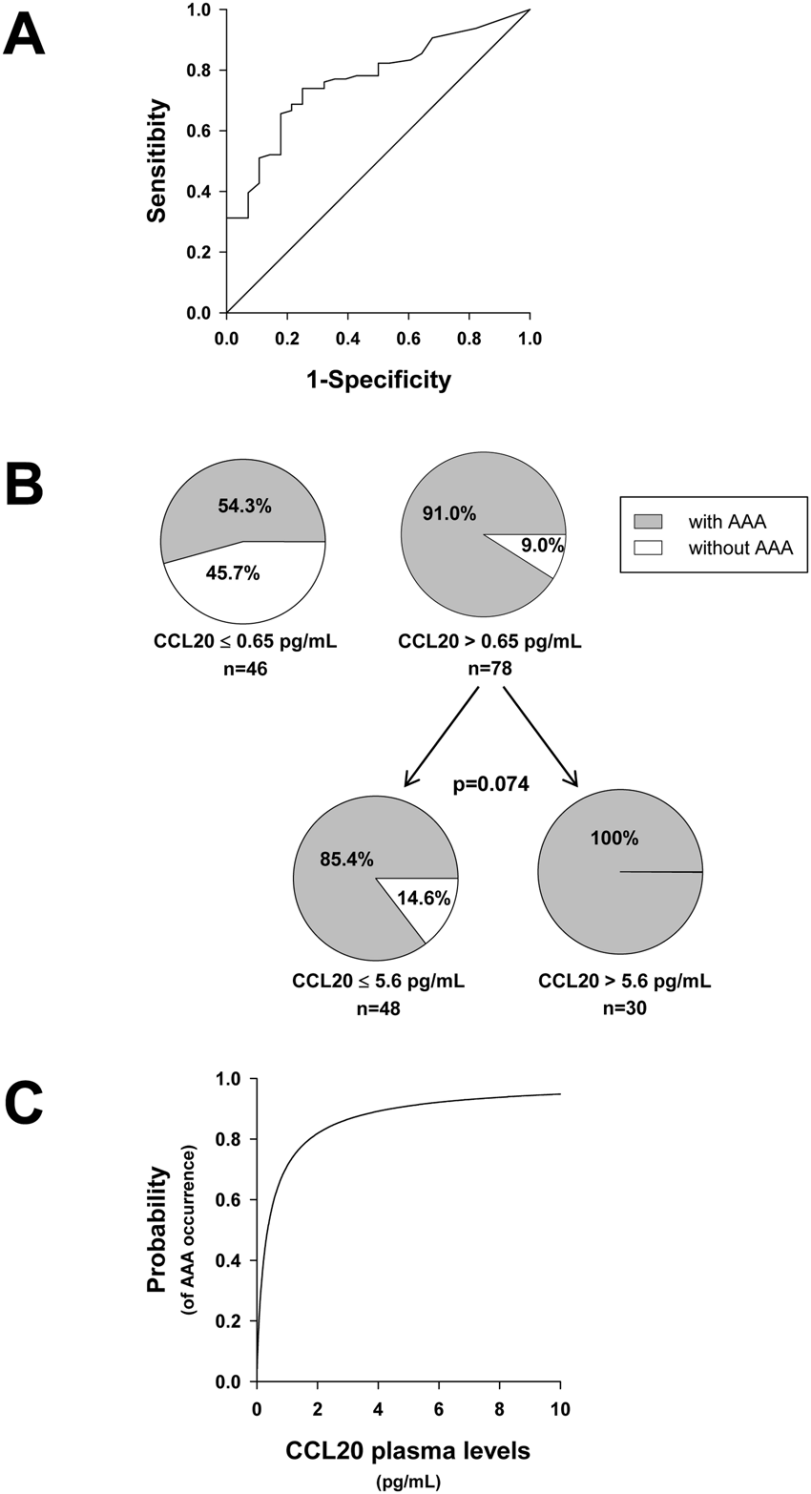
**(A**) Receiver-operating-characteristic (ROC) curve for CCL20 plasma levels in abdominal aortic aneurysm patients (AAA, n = 96). The area under the curve was 0.768 (CI 0.678–0.858; p < 0.001). **(B)** CART analysis classification regarding CCL20 plasma levels as independent variable and using AAA condition as the dependent variable; including all patients in the statistics. (**C)** Calculated probability from the equation Logit P = 0.901 + 2.006 ∗ log10 [CCL20] obtained in the Logistic Regression Analysis, considering CCL20 plasma concentration ([CCL20]) as independent variable and occurrence of AAA as the dependent dichotomous variable ([CCL20] coefficient p < 0.001).

Figure 1 shows that CCL20 plasma levels of AAA patients were significantly higher than those of AD and healthy individuals (control). Interestingly, there were no statistically significant differences in CCL20 plasma levels between AD patients and healthy individuals. In view of these results, we analysed CCL20 levels through the receiver operating characteristics (ROC) curve including AAA and AD patients. The area under the curve (AUC) for CCL20 was 0.768 (CI 0.678–0.858; p < 0.001) (Fig. 2A). Using AAA condition as the dependent variable, we performed classification and regression tree (CART) analysis and patients were classified into two categories, low (≤0.65 pg/mL) and high (>0.65 pg/mL) plasma level of CCL20 (Fig. 2B). The low plasma CCL20 group included 54.3% of AAA patients while the high CCL20 group was constituted by a significantly higher percentage of AAA patients (91%, p < 0.001). In turn, CART analysis divided the high CCL20 group into two categories ≤ 5.6 pg/mL and >5.6 pg/mL (Fig. 2B). Although the difference in the percentage of patients with AAA did not reach statistical significance (p = 0.074), it is noteworthy that the 100% of patients with CCL20 levels >5.6 pg/mL had AAA. Logistic regression analysis, considering [CCL20] as independent variable and occurrence of AAA as the dependent dichotomous variable, yields the following equation Logit P = 0.901 + 2.006 ∗ log_10_ [CCL20] ([CCL20] coefficient p < 0.001). Calculating the probability of having AAA for the two cut-offs we obtained 62.8% for 0.65 pg/mL and 91.7% for 5.6 pg/mL. The graph showing the calculated probability of AAA occurrence as a function of CCL20 plasma concentration is depicted in Fig. 2C.

**Figure 3:**
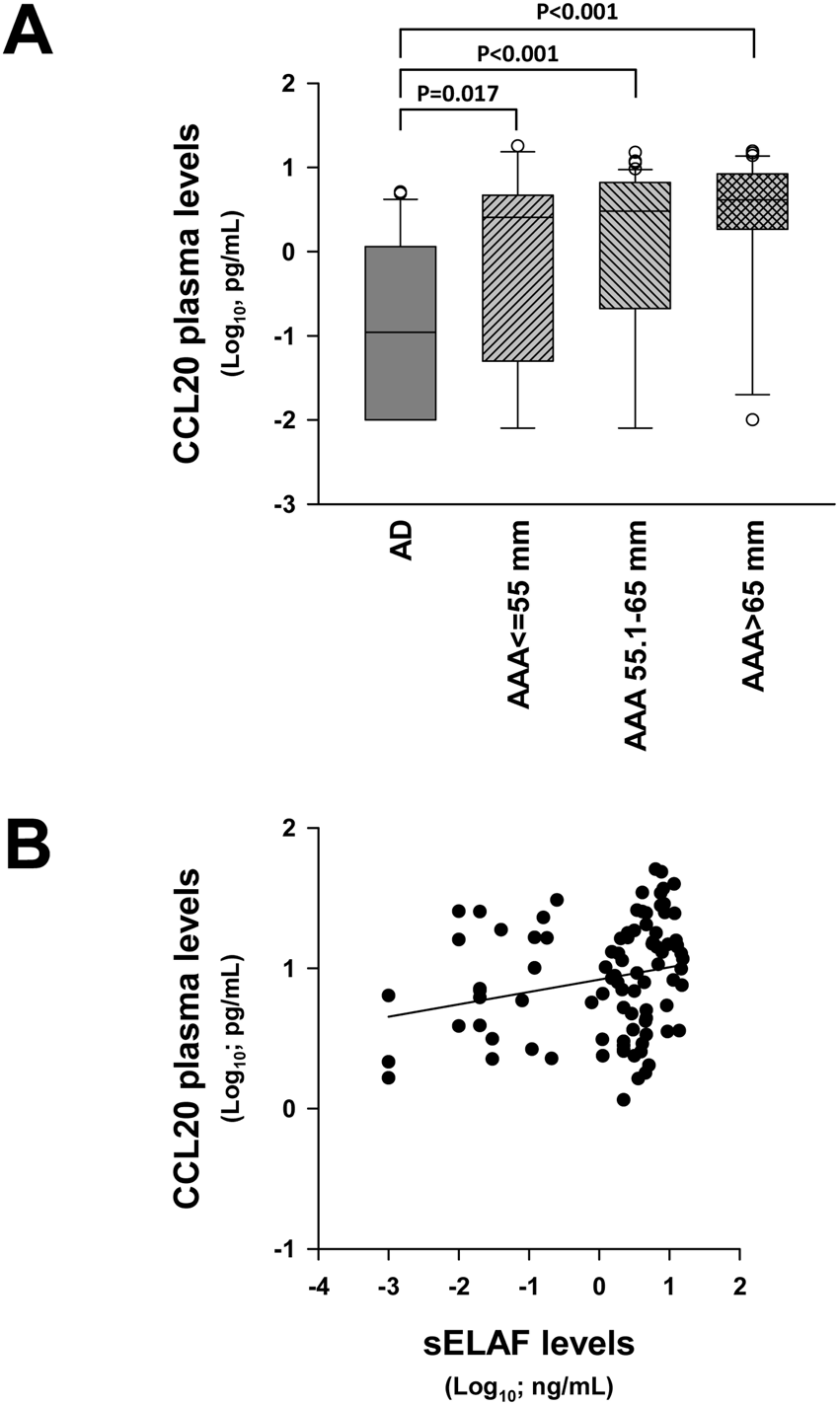
(**A)** CCL20 plasma concentration in abdominal aortic aneurysm patients stratified by MD (AAA) and patients with atherosclerotic disease without AAA (AD). No group fit normal distribution. **(B)** Statistical correlation of between CCL20 and sELAF in AAA samples.

We found no correlation between plasma levels of CCL20 with maximum aneurysm diameter (MD) (not shown). Patients were stratified into three groups according to the MD (MD ≤ 55 mm, 55 < MD ≤ 65 mm and MD >65 mm) and there were no significant differences among the three groups with regards to CCL20 plasma concentration. However, it is noteworthy that even the group of MD ≤ 55 mm had significantly higher level of CCL20 than AD patients (Fig. 3A).

Since plasma levels of soluble elastin fragments (sELAF) were associated with vascular wall deterioration, we analysed their plasma levels in AAA and AD patients. No significant differences were found in sELAF plasma levels between AAA and AD or correlation between them and MD (data nor shown). However, AAA patients exhibited a poor but significant correlation between CCL20 and sELAF plasma levels (Fig. 3B). When AAA patients were stratified by MD in the same manner as CCL20, no significant differences were observed among the three groups ≤ 55 mm, 55.1–65 mm and > 65 mm regarding sELAF.

### CCL20 expression in AAA tissues

Thereafter we quantified CCL20 local expression in AAA aorta tissue samples in terms of mRNA compared with normal aorta. The demographic data of the individuals included in mRNA analysis are shown in Table 3. There were significant differences in age, percentage of women, patients with dyslipemia, hypertension, PAD, ischemic heart disease, antiplatelet drug users and statin users between both groups. The influence of age on the CCL20 local mRNA levels was explored by multiple linear regression analysis after logarithmic transformation of CCL20 mRNA levels. Log_10_ of CCL20 was considered dependent variable and age and age^2^ as independent variables. No dependence of CCL20 local expression on age was observed in any of the groups, AAA or normal aorta (AAA: log_10_ CCL20 = −3.119 + 0.101 ∗ age −0.000758 ∗ age^2^, age coefficient p = 0.549, age^2^ coefficient p = 0.523, R = 0.099; Controls: Log_10_ CCL20 = −1.292 + 0.0297 ∗ age − 0.000326 ∗ age^2^, age coefficient p = 0.777, age^2^ coefficient p = 0.739, R = 0.124). No differences in CCL20 local mRNA levels between positive and negative individuals for women, dyslipemia, hypertension, PAD, ischemic heart disease, antiplatelet drug user and statin user in neither normal aorta nor AAA groups were also found. Then, none of these parameters were taken into account as confounding variables for the statistics.

**Table 3:** Demographics of individuals included in the local mRNA levels study.

	**AAA**	**Normal Aorta**	**p**
**n**	89	17	—
**Age (years)**	71.0 ± 6.6	57.5 ± 15.9	<0.001
**Women**	5.6	58.8	<0.001
**Aortic diameter (mm)**	65.0 ± 13.0	—	—
**Dyslipidemia**	60.7	17.6	0.003
**HTN**	71.9	17.6	<0.001
**Diabetes**	23.6	11.8	0.447
**Smokers**	27.0	11.8	0.305
**PAD**	49.4	0.0	<0.001
**BVD**	7.9	11.8	0.957
**IHD**	24.7	0.0	0.048
**COPD**	19.1	0.0	0.108
**Antiplatelet users**	59.6	11.8	<0.001
**Statin users**	68.5	5.9	<0.001
**NSAID users**	4.5	0.0	0.843
**Corticoid users**	4.5	0.0	0.843
**Immuno-supressors users**	4.5	0.0	0.843

Nominal variables are presented as %. Continuous variables are presented as mean ± SD. Due to the nature of normal aorta samples some of the clinical characteristics are not always recorded and infra-evaluation of them is probable. BVD, brain-vascular disease; COPD, chronic occlusive pulmonary disease; HTN, chronic hypertension; IHD, ischemic heart disease; PAD, peripheral artery disease.

**Figure 4:**
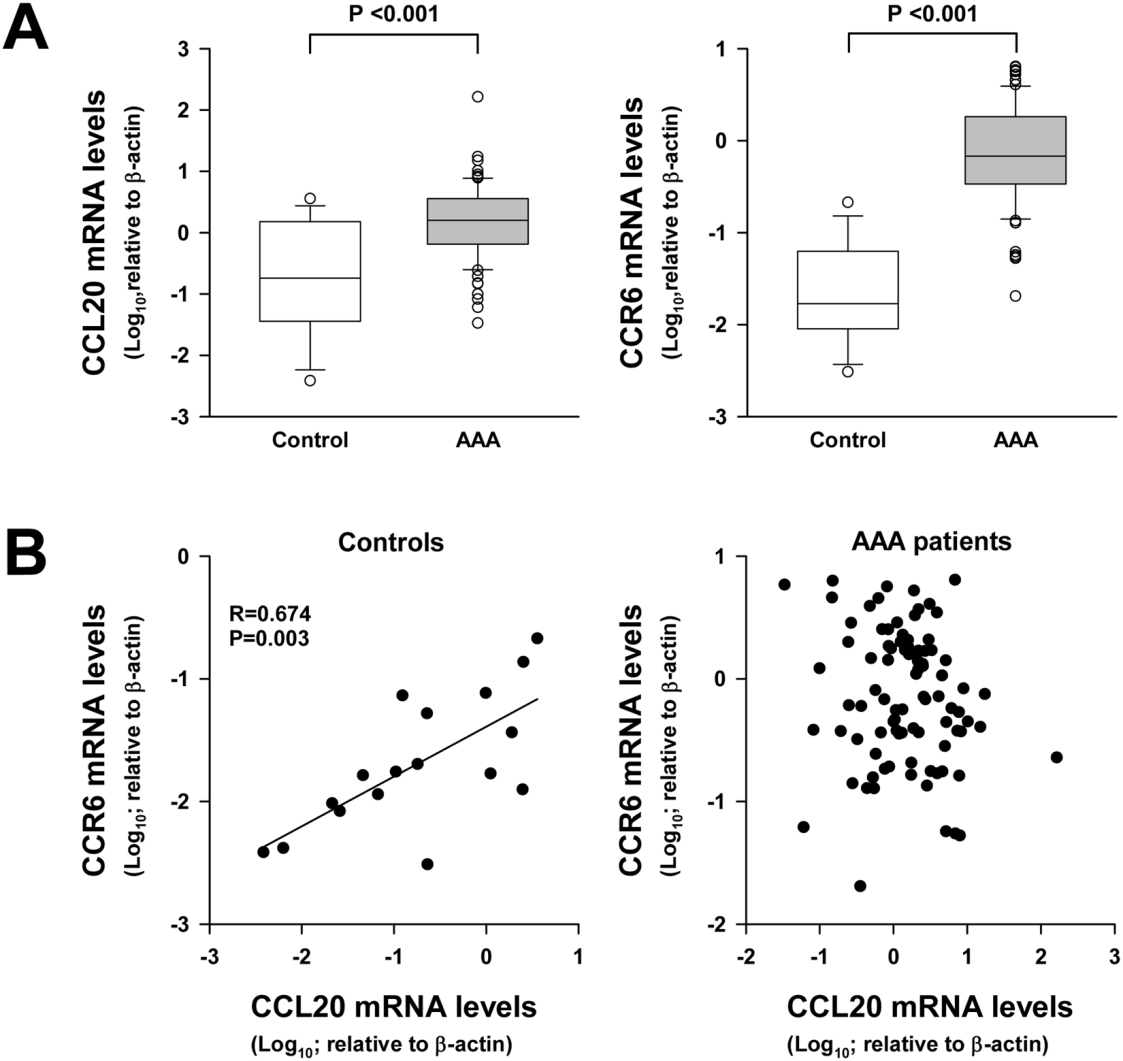
(**A)** CCL20 and its receptor (CCR6) transcription levels in normal aorta from healthy donors (control, n = 17) and abdominal aortic aneurysm lesions (AAA, n = 89). No group fit normal distribution. **(B)** Statistical correlation between CCL20 and its receptor transcription levels in normal aortas (control) and AAA samples.

**Figure 5:**
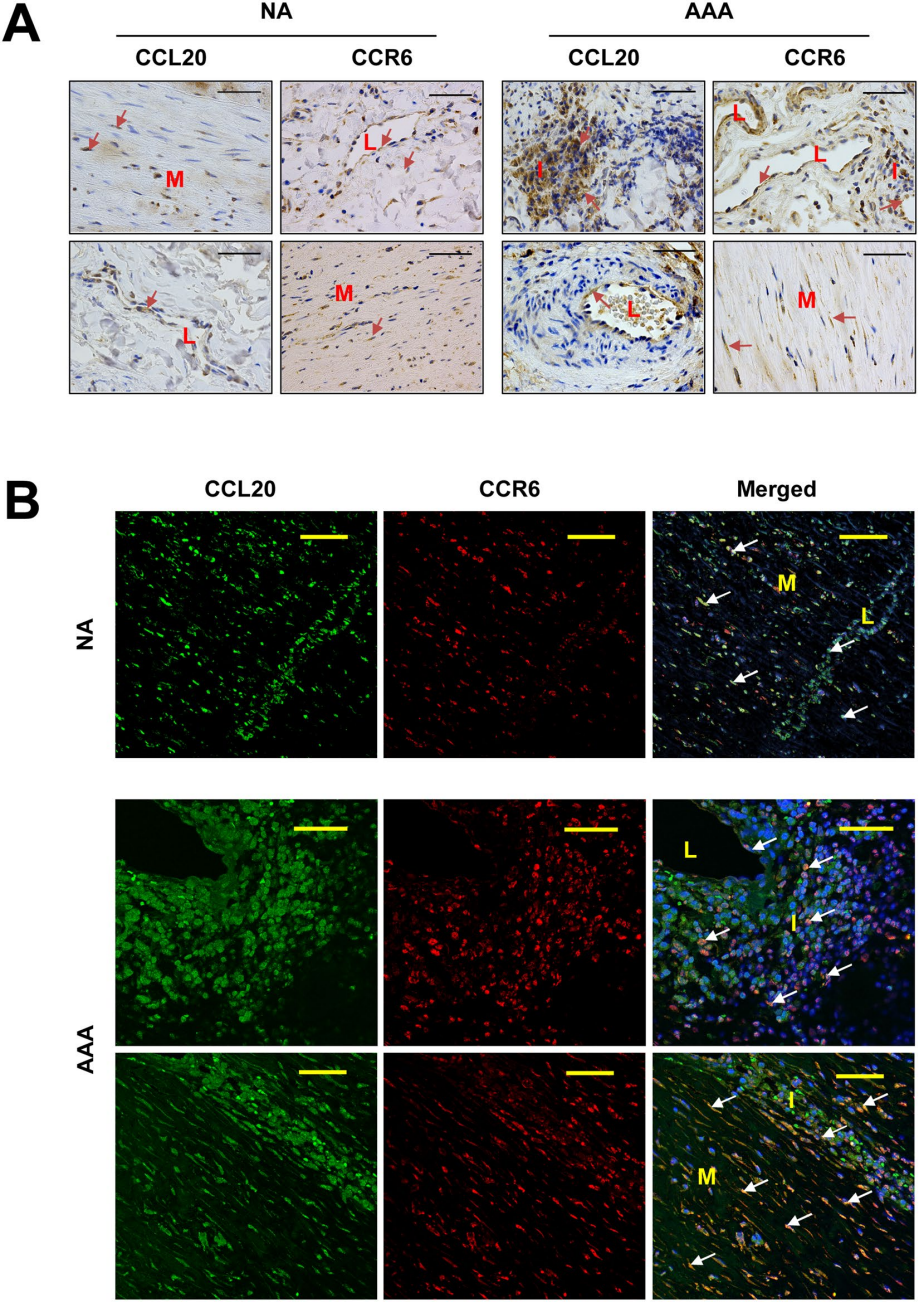
**(A)** Representative immunohistochemistry images of CCL20 and its receptor (CCR6) in normal aorta (NA) and in AAA samples. Arrows show some immunostained cells. (**B)** Representative immunofluorescent double staining for CCL20 and CCR6 in normal aorta (NA) and in AAA samples. Arrows show double immunostained cells. Bars are 50 µm; L, indicates the light of microvessels; I, indicates leukocyte infiltration areas; and M, indicates media layer.

Results depicted in Fig. 4A show that expression of both CCL20 and its receptor CCR6 were significantly higher in AAA samples than in normal aorta. Nevertheless, there was not statistical correlation between CCL20 and CCR6 mRNA expression in AAA samples. In contrast, there was a significant correlation between CCL20 and CCR6 local expression in normal aorta samples (R = 0674; p = 0.003, Fig. 4B). Immunohistochemical studies showed that CCR6 and CCL20 were widely expressed in normal aorta, in the medial layer and endothelial cells from the microvasculature in which they were often co-expressed (Fig. 5A and B). Further, in aneurysmatic samples CCL20 and CCR6 were also abundantly expressed by infiltrating leukocytes. However, and consistently with correlation studies, such co-expression did not occur in all infiltrating leukocytes and only few cells were clearly positive for both proteins (Fig. 5B).

## Discussion

AAA is a multifactorial disease with genetic and environmental risk factors. AAA is an immuno-inflammatory disease associated with atherosclerosis^5^ and, like other autoimmune pathologies, AAA progressively degenerates until the destruction of normal tissue. CCL20 has been associated with several autoimmune diseases^17,24–26^ and this fact led us to investigate the association between CCL20 and AAA.

Here, we comparatively investigated the expression of the CCL20/CCR6 system in AAA as well as in non-AAA atherosclerotic patients and healthy individuals. Our results show that circulating CCL20 is significantly increased in AAA when compared with healthy individuals. Most importantly, CCL20 is also increased when compared with individuals with atherosclerotic vascular pathology without AAA. It should be highlighted that this is a unique feature of CCL20, not observed for other cytokines also increased in the AAA, suggesting that CCL20 is particularly relevant in patients suffering from AAA.

We must point out that unlike other authors^27^, we found no statistical correlation between the aortic diameter and plasma levels of any of the cytokine tested (results not shown) in aneurysmatic patients. One possible explanation for this discrepancy could be that when we analyzed the statistical correlations of MD and plasma levels, only patients with AAA were taken into account. Particularly, we did not find a statistical correlation between the aortic maximum diameter and plasma levels of CCL20. If we assume that the aortic diameter somehow represents the chronology of AAA and considering that CCL20 is significantly higher even in patients with the smallest diameter, our results suggest that CCL20 is increased from the early stages of the disease. The lack of correlation between plasma concentration of CCL20 and the aortic diameter is not surprising since, in our view, plasma levels of a chemokine reflect a systemic immune situation while the aortic diameter might reflect the evolution of the injury. *A priori*, there is no reason in our view, to suppose a correlation between systemic levels of a protein involved in the immune response measured in a particular moment with the degree of evolution of a related injury. Presumably, the extent of injury depends on how long tissue states under inflammatory condition rather than at a given time concentration of a specific immune mediator in that tissue and still less likely on its plasma levels. Indeed, some inflammatory mediators reach a maximum in patients with intermediate MD and then decreases at higher MD^28–31^. Another issue is the relationship of plasma levels with the rate of progression of the injury that is probably strictly related to the intensity of the immune response. In this regard we have not measured the AAA growth rate, which is a limitation of the present study. However, we found a significant correlation between CCL20 and sELAF, which is related to the degradation of the vascular wall and exhibit pro-inflammatory activity^32^.

In addition, we observed that CCL20 levels are a sensitive marker for AAA, CCL20 high levels are associated with AAA presence. The statistical study shows that plasma concentrations above 5.6 pg/mL imply a likelihood of AAA higher than 90%.

Both atherosclerosis and AAA are diseases characterized by a great inflammatory infiltrate. However, our results reveal that significant differences exist regarding factors involved in the recruitment of immune-inflammatory cells to the aortic wall and their local activation between these two disorders.

We also report a significant CCL20/CCR6 up-regulation in AAA tissues compared to normal aortic tissues. It is noteworthy that in normal aortas local CCL20 expression correlates with the expression of its receptor CCR6, which is not the case in AAA samples. Immunohistochemical studies revealed that both proteins were expressed by vascular cells, while only part of the massive inflammatory infiltrate in AAA samples co-expresses CCL20 and its receptor. This could explain why correlation between CCL20 and its receptor could only be observed in normal aorta.

In summary, we showed that plasma concentration of CCL20 was significantly higher in patients with AAA when compared with healthy individuals and, more importantly, with patients having atherosclerotic pathology without AAA. The statistical analysis shows that CCL20 plasma levels predict with high sensitivity the presence of AAA. CCL20 was also overexpressed in patients with AAA in terms of local transcript levels compared with healthy individuals. The role and the nature of the immune-mediated mechanisms in AAA have recently started to be addressed^4,5^. The present study shows an association of CCL20 with AAA, but the role of CCL20 in the pathogenesis and progression of AAA still remains uncertain. To asses this point, further research is needed.

## Material and Methods

### Patients

The study was approved by the Hospital de la Santa Creu i Sant Pau Ethics Committee (13/103/1491), and patients gave written informed consent prior to surgery. The study conformed to the principles of the Declaration of Helsinki. Clinical outcomes were taken from the clinical database.

We defined two groups of patients as follows, patients with AAA and patients with atherosclerotic disease without AAA (AD). Inclusion criteria of AAA group were patients undergoing elective open or endovascular repair for atherosclerotic AAA. Exclusion criteria were patients with pseudoaneurysms, or infectious or inflammatory aneurysms. When possible an infrarenal aorta biopsy was taken during the intervention. All AAA patients underwent surgery at Hospital de la Santa Creu i Sant Pau. AD group was constituted by patients admitted during the same period in our centre with ischemic pathology diagnosis of either lower limbs or carotid territory. Patients with aortic or peripheral aneurismal pathology were excluded from this group. Blood was collected before anaesthesia in the operating room. A sample of 10 mL of peripheral blood was collected from the individuals in heparin-containing tubes. It was centrifuged immediately and plasma aliquoted and frozen at −80 °C until CCL20 analysis.

### Tissue samples

Biopsies were systematically performed on the anterolateral wall of the remaining mid-infrarenal aortic wall after the exclusion and prosthetic replacement of AAA, at the level of the inferior mesenteric artery. Intraluminal thrombi, if present, were separated before the aorta biopsy was taken. Normal aortas (Controls) were obtained from healthy aorta from multiorgan donors and samples were also taken from the mid-portion of the infrarenal abdominal aorta at organ harvest. Biopsies were processed immediately, a portion of each sample was placed in RNAlater solution (Qiagen GmbH, Hilden, Germany) and stored for 24 hours at 4 °C before long-term storage at −80 °C until further processing for RNA isolation.

### Risk factors

The definition of the risk factors used in this study were: diabetes mellitus: glycated haemoglobin >5.8% or use of oral antidiabetic drugs or insulin; arterial hypertension: systolic blood pressure ≥ 140 mm Hg, diastolic blood pressure ≥ 90 mm Hg or use of antihypertensive medication; dyslipidemia: a total cholesterol >6.2 mmol/L, LDL cholesterol >4.13 mmol/L, HDL cholesterol < 1 mmol/L or triglycerides >1.65 mmol/L; smoking was categorized into 2 groups: smokers: smokers and ex-smokers stopped smoking < 1 year, and non-smokers: never-smoked and ex-smokers stopped smoking >1 year; COPD: FEV1/FVC < 0.7, brain-vascular disease: history of stroke, transient ischemic attack or major neurological deficit; ischemic heart disease: history of myocardial infarction, angina pectoris or previous coronary intervention; peripheral artery disease: including any stage of Fontaine classification or previous revascularization intervention or ischemic amputation.

### RNA extraction and mRNA analysis

Tissues were homogenized in the FastPrep-24 homogenizer and Lysing Matrix D tubes (MP Biomedicals, Solon, OH). RNA was extracted using Trizol (Invitrogen, Carlsbad, CA) following the manufacturer’s instructions. cDNA was prepared by reverse transcribing 1 µg RNA with the High-Capacity cDNA Archive Kit with random hexamers (Applied Biosystems, Foster City, CA). mRNA levels of CCL20 and CCR6 were studied by real-time PCR in an ABI Prism 7900HT using pre-designed validated assays (TaqMan Gene Expression Assays; Applied Biosystems) and universal thermal cycling parameters. Relative expression was expressed as transcript/β-actin ratios.

### Plasma measurements

CCL20, IL1-β, RANTES, VEGF, MPO, IL-8, soluble ICAM, IP-10, CCL20, TNFα, MMP-9, IL-2, IGFBP-1, soluble Fractalkine, IL-10 and soluble TWEAK protein levels in plasma were analyzed in a Luminex using xMAP® technology (Millipore Corporation, Billerica, MA), and sELAF levels were analyzed using ELISA (MyBiosource, San Diego, CA, USA) following manufacturer’s instructions.

### Immunohistochemistry

Immunohistochemical studies were performed using a goat polyclonal antibody against human CCL20 (diluted 1:100) and a mouse monoclonal anti human CCR6 (diluted 1:200) both from R&D Systems (Minneapolis, MN). Three-micrometer sections of paraffin-embedded tissue samples were stained in a Dako Autostainer Link 48 using the Dako EnVision Flex Kit. Diaminobenzidine was used as chromogen.

### Double immunofluorescence

For co-localization studies, double immunofluorescence staining was performed. After deparaffinization, rehydration and antigen retrieval, blocking for unspecific binding was performed for 1 hour at room temperature. A mixture of two primary antibodies, CCL20 and CCR6, in 1% BSA was then applied for 1 hour at room temperature, After washing with PBS, the sections were incubated with a mixture of two secondary antibodies in 1% BSA for 1 hr at room temperature in dark (Alexa Fluor 488 conjugated rabbit-antigoat IgG (H + L) and biotinylated horse-antimouse IgG (H + L) both diluted 1/250, Invitrogen, Life Technologies Co, Eugene, OR) followed by 45 minutes incubation with Streptavidin protein Alexa Fluor 594. Nuclei were counterstained with Hoechst 33258 for 10 minutes. As a negative control, sections were incubated omitting primary antibodies. Samples were then mounted with ProLong Gold antifade reagent (Molecular Probes, Life Technologies Co). Images were obtained using an SP5 Leica confocal microscope.

### Statistical analysis

SPSS and Sigma-Plot software were used for statistical analysis. To evaluate the statistical differences in the demographic variables between two groups the t-test was used for those continuous variables that fit a normal distribution and Mann-Whitney U test for those continuous variables that did not have a normal distribution. To observe differences in the demographic dichotomous variables between two groups we used z-test. Kruskal-Wallis One Way Analysis of Variance on Ranks was used to compare CCL20 levels in more than two groups since they did not fit normal distribution. Spearman Rank Order Correlation was used for correlation between variables. Multiple linear regression was used to study the association of CCL20 plasma levels with quantitative demographic variables and multiple logistic regression was used to study the likelihood of the occurrence of AAA as a function of CCL20 plasma levels. We used ROC curves to evaluate capacity of CCL20 to discriminate between AAA and AD patients. Continuous value parameters were analyzed using a classification and regression tree (CART) analysis, considering AAA condition as a dependent variable. The CART analysis split the continuous data into segments that were as heterogeneous as possible, according to the dependent variable. A p value < 0.05 was considered significant.
